# The virtual circular genome model for primordial RNA replication

**DOI:** 10.1261/rna.077693.120

**Published:** 2021-01

**Authors:** Lijun Zhou, Dian Ding, Jack W. Szostak

**Affiliations:** 1 Howard Hughes Medical Institute, Department of Molecular Biology and Center for Computational and Integrative Biology, Massachusetts General Hospital, Boston, Massachusetts 02114, USA; 2 Department of Genetics, Harvard Medical School, Boston, Massachusetts 02115, USA; 3 Department of Chemistry and Chemical Biology, Harvard University, Cambridge, Massachusetts 02138, USA

**Keywords:** origin of life, nonenzymatic replication, protocell, RNA, oligonucleotides

## Abstract

We propose a model for the replication of primordial protocell genomes that builds upon recent advances in the nonenzymatic copying of RNA. We suggest that the original genomes consisted of collections of oligonucleotides beginning and ending at all possible positions on both strands of one or more virtual circular sequences. Replication is driven by feeding with activated monomers and by the activation of monomers and oligonucleotides in situ. A fraction of the annealed configurations of the protocellular oligonucleotides would allow for template-directed oligonucleotide growth by primer extension or ligation. Rearrangements of these annealed configurations, driven either by environmental fluctuations or occurring spontaneously, would allow for continued oligonucleotide elongation. Assuming that shorter oligonucleotides were more abundant than longer ones, replication of the entire genome could occur by the growth of all oligonucleotides by as little as one nucleotide on average. We consider possible scenarios that could have given rise to such protocell genomes, as well as potential routes to the emergence of catalytically active ribozymes and thus the more complex cells of the RNA World.

## INTRODUCTION

The idea that the genomes of the first protocells could have been replicated by purely nonenzymatic processes is attractive because, in the absence of any prior evolutionary steps, the emergence of effective macromolecular catalysts such as ribozyme replicases seems implausible. Even if a ribozyme that was small enough and active enough to mediate genomic replication was formed by chance within a protocell, the initiation of exponential amplification would require two copies of the ribozyme sequence—one to act as an enzyme, and the other acting as the template to be copied (Orgel 1998; Szostak et al. 2001). It is highly unlikely that two copies of a replicase would arise independently within the same protocell. The alternative, and more plausible scenario, is that one copy was formed by chance, and was then copied chemically to produce + and − strands. However, if nonenzymatic copying of a ribozyme sequence is possible, then cycles of replication might also be possible, in which case nonenzymatic replication could allow for the initial emergence and subsequent evolution of diverse ribozymes in addition to a replicase (Szostak 2012). Thus, the nonenzymatic replication of primordial RNA genomes could have played a major role in initiating the emergence of an RNA World.

Although the idea of nonenzymatic RNA replication is compelling, the development of a coherent model for the nonenzymatic replication of a primordial RNA genome is still problematic (Orgel 2004; Szostak 2012; Joyce and Szostak 2018). One difficulty stems from the view that sequences of 20–30 nt in length or longer must be replicated to enable ribozyme emergence and evolution. Although considerable progress has been made in uncovering more efficient chemical processes for template-directed primer extension and ligation (Jauker et al. 2015; Kervio et al. 2016; Prywes et al. 2016; Li et al. 2017; Zhou et al. 2020a), no means for the effective copying of RNA sequences of this length has yet been found. Even if copying could be made more efficient, replication requires a means of copying the copies. However, strand separation becomes increasingly difficult as oligonucleotide length increases, and even after strand separation, complementary oligonucleotides tend to renature very rapidly, preventing copying of the copies. These issues recently led us to propose that the nonenzymatic replication of long oligonucleotides might not be necessary, if active ribozymes could be assembled as needed by the ligation of much shorter oligonucleotides that could actually replicate (Zhou et al. 2020b).

**FIGURE 1.**
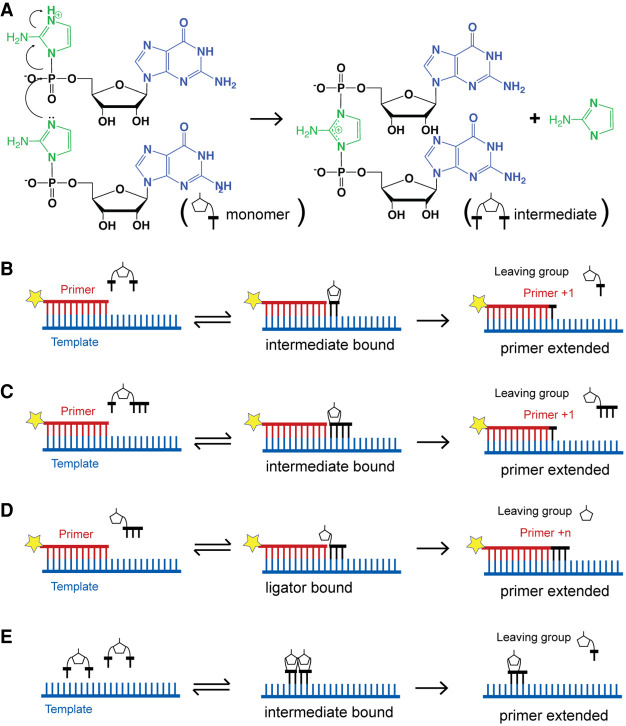
Primer extension and oligonucleotide ligation: the elementary reactions of nonenzymatic RNA replication. (*A*) Primer extension begins with the reaction of one activated nucleotide with a second activated nucleotide (or oligonucleotide). Here, two guanosine nucleotides activated with 2-aminoimidazole are shown reacting to form the 5′–5′-linked imidazolium-bridged dinucleotide intermediate. (*B*) The intermediate binds to a template oligonucleotide downstream from a primer oligonucleotide. Attack of the 3′-hydroxyl of the primer on the adjacent phosphate of the intermediate results in primer extension by one nucleotide, with release of an activated nucleotide as the leaving group. (*C*) An imidazolium-bridged intermediate formed by reaction of an activated monomer with an activated oligonucleotide results in faster and more efficient primer extension. (*D*) Binding of an activated oligonucleotide to a template, downstream from a primer, can result in ligation. This process is normally slow and inefficient but can be accelerated by organocatalysts such as N-alkyl imidazoles. (*E*) The synthesis of new oligonucleotides may be initiated when two imidazolium-bridged dinucleotides bind next to each other on a template strand. The upstream dinucleotide acts as a primer, attacking the downstream intermediate, giving rise to an imidazolium-bridged trinucleotide.

**FIGURE 2.**
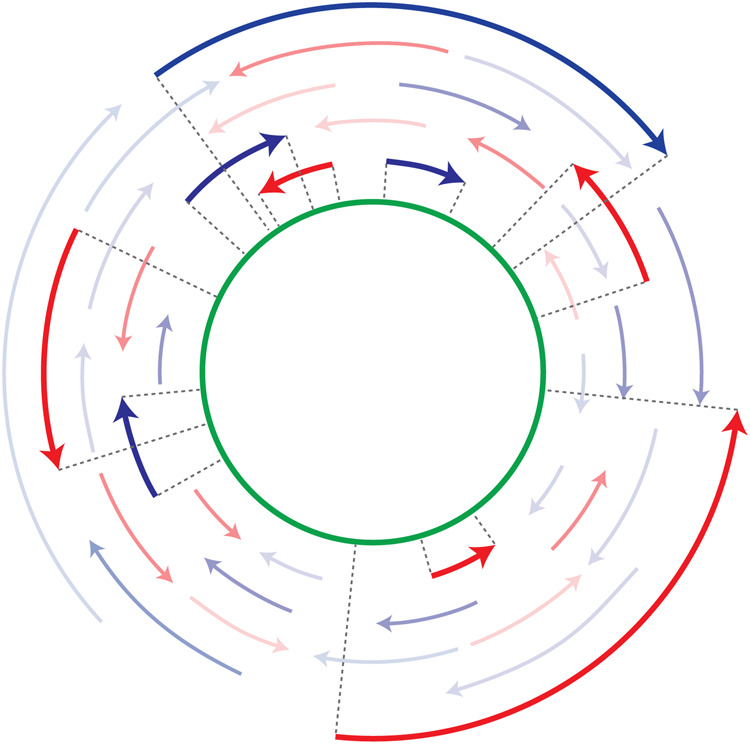
Schematic of the virtual circular genome model. (Green circle) The virtual circular genome is an abstract circular sequence that need not correspond to any actual oligonucleotide. (Blue and red arrows) A subset of the collection of oligonucleotides beginning and ending at every position on the virtual circular genome. A few of the many oligonucleotides are shown as bold colored arrows, with dotted lines showing how they map onto the sequence of the virtual circular genome.

While the nonenzymatic replication of shorter oligos seems more plausible than the replication of longer sequences, several problems remain (Szostak 2012). The most obvious is that in most model systems for the study of template copying chemistry, it is the extension of a defined primer that is actually monitored. Of course, in a primordial situation, no defined primers would have been available. How could a small genomic sequence be replicated without a continuous input of defined primers that would serve to maintain each end of the linear sequence? A related problem is the so-called “last base addition problem” (Wu and Orgel 1992). In primer extension experiments, copying of a good template (e.g., a C-rich oligo) proceeds well until the last base, which is filled in at a very slow rate. This is because primer extension occurs primarily through reaction of the primer with an imidazolium-bridged dinucleotide intermediate derived from the reaction of two activated monomers with each other (Fig. 1; Kervio et al. 2016; Walton and Szostak 2016). The imidazolium-bridged dinucleotide normally binds to the template by two Watson–Crick base-pairs (Walton and Szostak 2016, 2017; Zhang et al. 2018; Walton et al. 2019). This is not possible when copying the last base of the template, and as a result the rate of primer extension slows dramatically. Further, in a prebiotic situation, template copying might frequently initiate at internal sites such that ligation events would be required to stitch together partial template copies (Szostak 2011, 2012). However, the initiation of oligonucleotide synthesis with a 5′–5′ pyrophosphate linked dinucleotide, or simply an unphosphorylated nucleoside would generate 5′ ends that cannot be ligated and would thereby prevent the synthesis of a full-length template copy. All these problems could be avoided, in principle, if the genome is a single-stranded circular RNA molecule because short, random oligonucleotide sequences could initiate template copying at any position. However, conversion of a short single-stranded circle to a double-stranded circle would be difficult because short dsRNA circles are highly strained due to the high bending energy of dsRNA. Moreover, copying of a circular template still requires efficient copying of long sequences, as well as cleavage and recircularization of the product strands, as seen in virion RNAs where ribozymes are used to catalyze these reactions (Flores et al. 2011). Thus, primordial genomes in the form of covalently closed circular RNA molecules seem unlikely. Here we propose a potential solution to the above problems: a “virtual” circular genome represented by all possible fragments from both strands, but with no actually circular genomic molecules existing (Fig. 2).

## HYPOTHESIS

**FIGURE 3.**
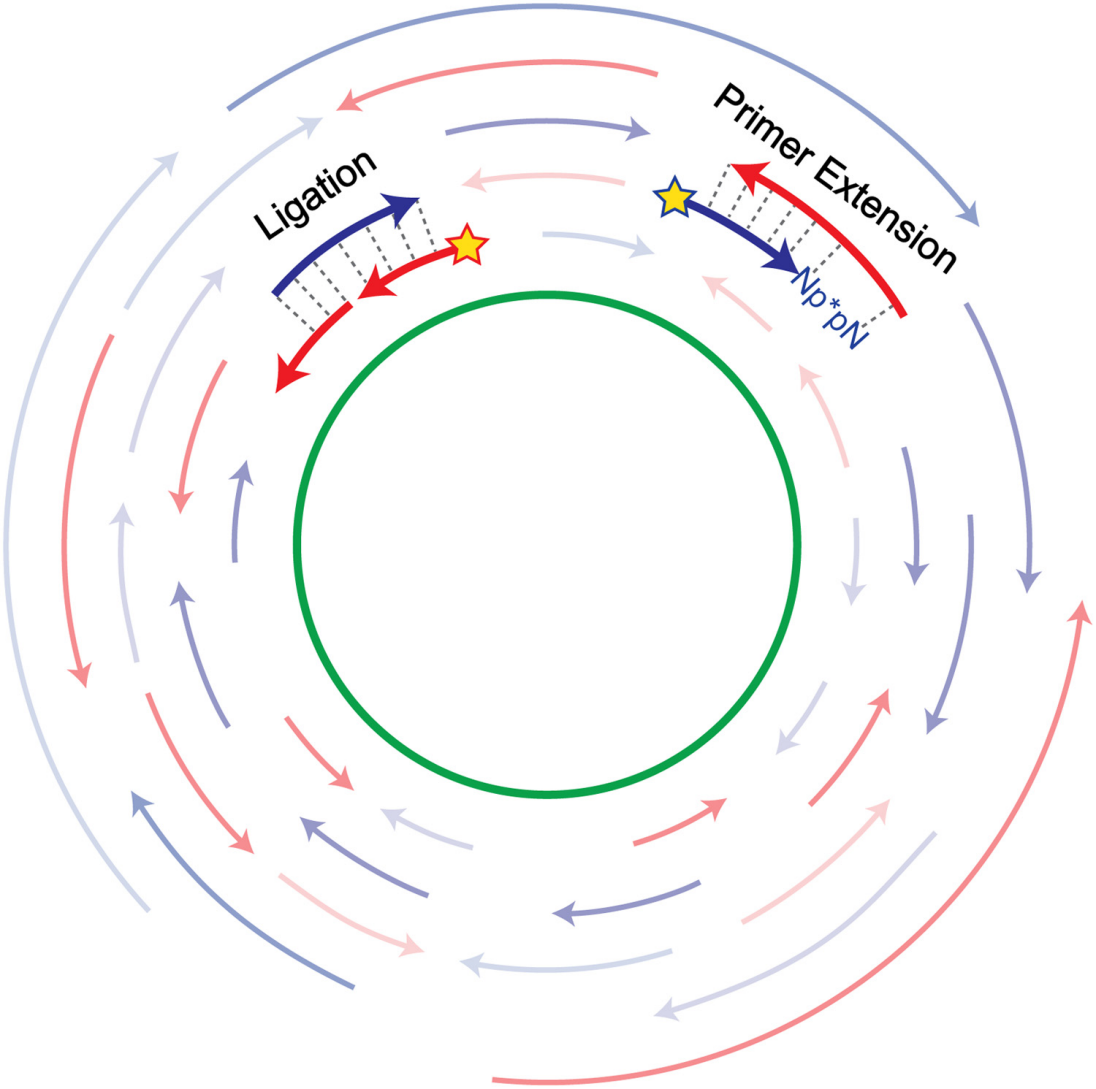
Oligonucleotides can grow by primer extension or by ligation. Such reactions could be followed experimentally by labeling a specific oligonucleotide, for example with a 5′-^32^P (represented as a star). (*Right*) A specific labeled oligonucleotide reacts with an imidazolium-bridged dinucleotide resulting in template-directed primer extension by one nucleotide. (*Left*) A specific labeled oligonucleotide undergoes template-directed ligation with an adjacent oligonucleotide.

**FIGURE 4.**
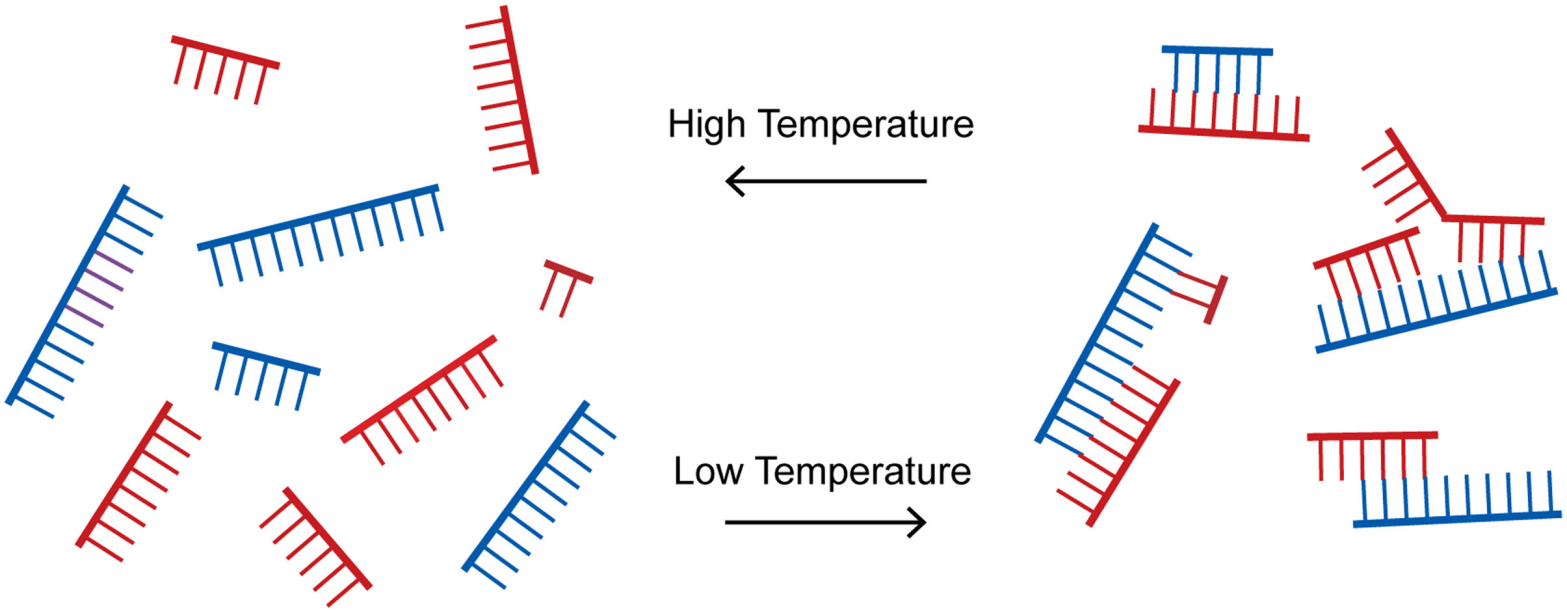
Cycling between separated oligonucleotides and diverse annealed configurations. A brief exposure to high temperatures (and/or low salt or extremes of pH) leads to strand separation. Subsequent return to annealing conditions results in the formation of a large number of kinetically trapped partially base-paired configurations.

We propose that primordial protocellular genomes consisted of sets of oligonucleotides with all (or most) possible starts and stops on both strands of one or more virtual circular sequences (Fig. 2). Genome replication would be driven by the input of new monomers and perhaps dimers and trimers. In the presence of an appropriate activation chemistry these monomers and oligonucleotides would have become activated, for example as 5′-phosphoro-imidazolides. Such activation would have enabled oligonucleotide elongation by both template-directed primer extension and ligation (Fig. 3). Thermal cycles that repeatedly shuffled and rearranged the secondary structures formed by base-pairing between oligonucleotides after cooling (Fig. 4) would have allowed for continued template-directed oligonucleotide growth. We suggest that the shortest oligos would have been the most abundant, with longer oligos becoming progressively less abundant. A surprising consequence of such a concentration versus length gradient is that average oligonucleotide growth by as little as one nucleotide could result in replication of the entire genomic ensemble (Table 1).

**TABLE 1.**
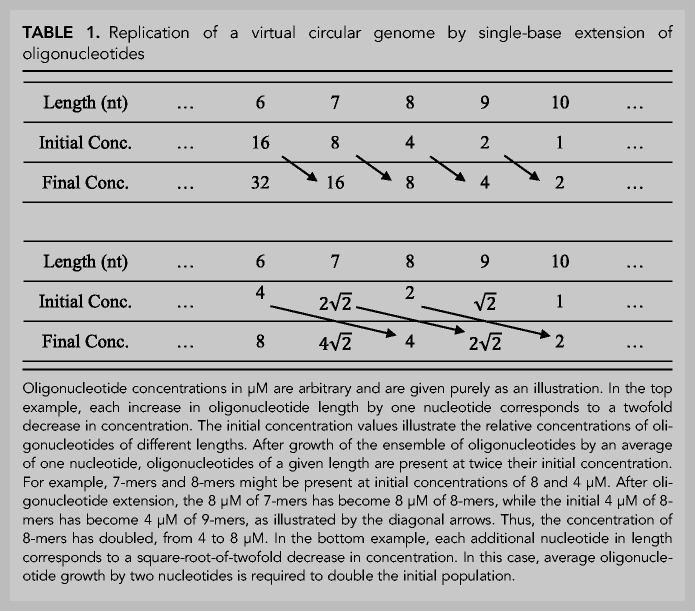
Replication of a virtual circular genome by single-base extension of oligonucleotides

Below we discuss the potential inputs of nucleotides, oligonucleotides and activation chemistries required for the replication of primordial genomes in the context of our model. We then discuss ways in which virtual circular genomes might arise, and factors that might affect their stable propagation. We then consider how such primitive genomes might evolve in response to competitive selection pressures, potentially enabling the emergence of selectively advantageous ribozymes within protocells. Finally, we discuss possible ways to experimentally test this hypothetical mode of nonenzymatic RNA replication.

## REPLICATION OF THE VIRTUAL CIRCULAR GENOME

### Inputs to the replication process

The most effective inputs of material and energy would be activated 5′-phosphorylated mono- or oligonucleotides, which would contribute to the growth of genomic oligonucleotides by primer extension and ligation, respectively (Fig. 1B–D). At present, the most plausible known 5′-phosphate activating group is 2-aminoimidazole (2AI) (Li et al. 2017), which can be synthesized together with the pyrimidine nucleoside precursor 2-aminooxazole (Fahrenbach et al. 2017). Activation of monomers or oligomers with 2AI has been demonstrated by the isocyanide pathway (Mariani et al. 2018b), a variant of which leads to highly efficient synthesis of 5′–5′ imidazolium-bridged dinucleotides (Zhang et al. 2020)—the dominant substrates for nonenzymatic template-directed primer extension.

### Initiation of oligonucleotide synthesis

The pioneering studies of Orgel showed that the template directed synthesis of oligonucleotides from activated monomers can occur in the absence of primers (Weimann et al. 1968). This process was always somewhat puzzling given the very weak binding of mononucleotides to a template strand. We now suggest that much of this synthesis occurs when two imidazolium-bridged dinucleotides bind next to each other on a template strand. The template-binding of imidazolium-bridged dinucleotides is much tighter than monomer binding because both nucleobases can take part in Watson–Crick pairing with the template, and because the imidazolium bridge contributes a positive charge. In this scenario the upstream dinucleotide acts as a primer and attacks the downstream dinucleotide to generate a trinucleotide, that could then continue to elongate by either primer extension or ligation (Fig. 1E). Subsequent hydrolysis would yield an oligonucleotide with either a 5′-phosphate or a 2AI-activated 5′-phosphate. Given an input of activated monomers, this process may be the primary driver of the synthesis of new oligonucleotides.

A second process that could contribute to the initiation of oligonucleotide synthesis is the spontaneous nontemplated oligomerization of activated monomers in solution, on mineral surfaces or in concentrated eutectic phases. Such processes are known to generate short oligonucleotides with random sequences (Ferris et al. 1996; Ertem and Ferris 1997; Monnard et al. 2003; Monnard and Szostak 2008). Di- and trinucleotides could form spontaneously in solution inside protocells, or could form outside protocells by any of the above processes, followed by diffusion across the membrane to the inside (Mansy and Szostak 2008; O'Flaherty et al. 2018). Once inside, they could become elongated by primer extension or by templated ligation. Longer oligonucleotides that formed outside the protocell would be unable to cross the membrane barrier and therefore be unable to contribute to genomic replication.

### Processes contributing to elongation

**FIGURE 5.**
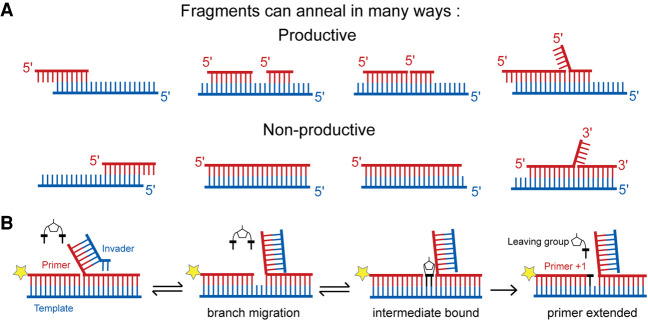
Some of the many possible annealed configurations of the oligonucleotides in a protocell. (*A*) A fraction of the annealed configurations would allow primer extension or ligation events to occur (*top*), while others would not (*bottom*). (*B*) Annealing of an invader strand to a 5′-overhang can transiently open up a template region, converting a nonproductive configuration to a productive configuration and thereby allowing primer extension to occur.

Under conditions favorable to base-pairing (low temperatures, high salt, moderate pH) a large number of different kinetically trapped base-pairing configurations would be expected to arise (Fig. 4). A fraction of these metastable states would allow a complementary imidazolium-bridged dinucleotide to bind to a template strand downstream from an oligonucleotide, which could then act as a primer and become extended by one nucleotide (Fig. 5A). In some cases, binding of a bridged dinucleotide would require opening of the template by the toehold and branch migration action of an invader strand (Fig. 5B; Zhou et al. 2019). An additional fraction of the base-paired configurations would bring an oligonucleotide next to an activated downstream oligonucleotide, with the consequent potential for ligation to occur.

Because a large fraction of base-paired configurations would not be compatible with either primer extension or ligation, the continued growth of all oligos would require repeated shuffling of their base-paired configurations (Fig. 4). Such rearrangements could be mediated by repeated cycles of full or partial denaturation by thermal or other environmental fluctuations such as salt concentration or pH (Ricardo and Szostak 2009; Mariani et al. 2018a; Ianeselli et al. 2019; Damer and Deamer 2020). Following each excursion to a less base-paired state, a return to favorable annealing conditions would allow a new subset of productive pairings to form. The number of such cycles that would be required to enable complete replication (i.e., doubling) of the initial population would depend on many variables, including the average lifetime of different productive pairing configurations, and the rates of primer extension and ligation reactions, both of which would in turn depend upon conditions such as temperature and divalent metal ion concentrations. Structural variants of RNA with stronger base-pairing (e.g., 2-thio-U:A base-pairs instead of A:U base-pairs) (Heuberger et al. 2015) might lead to longer lifetimes of productive configurations, thereby enhancing the efficiency of copying. On the other hand, because many paired configurations would involve relatively short regions of complementarity, spontaneous unpairing and pairing events might lead to an appreciable level of shuffling events that could either add or remove productive configurations. Such spontaneous shuffling events might be favored by a version of RNA with weaker base-pairing (e.g., with I:C base-pairs in place of G:C base-pairs) (Kim et al. 2018; Wright et al. 2018). In either case, over time, such rearrangements would be expected to converge on the overall thermodynamic minimum of full base-pairing, thus decreasing the fraction of productive pairings with time, and requiring another environmental fluctuation to allow for continued copying chemistry.

### Processes terminating oligonucleotide growth

As oligonucleotides grow longer, at least three processes are likely to decrease or stop continued elongation. First, above a certain length, complementary oligonucleotides may anneal to form stable RNA duplexes that are difficult or impossible to thermally denature. Completely stable duplexes would be dead-end products that no longer contribute to either genomic replication or the assembly of useful products such as ribozymes. Such duplexes would be serially diluted during protocell growth and division, and hence would be expected to approach a steady state concentration. As long as that steady state concentration is relatively low, such duplexes would be unlikely to destabilize the protocell or interfere with genomic replication.

Second, any pool of activated ribonucleotides generated by prebiotic chemistry would almost certainly also contain other nucleotides such as for example arabino- or threo-nucleotides. The incorporation of an arabino-nucleotide into a growing oligonucleotide during primer extension is essentially a chain terminating event (Kim et al. 2020). Such events would be fatal if genome replication required continuous end-to-end copying of a template strand. In contrast, in the virtual circular genome model, such events would simply generate a fraction of oligonucleotides that are no longer growing, but which can still play useful roles as templates, invaders for strand displacement synthesis (Zhou et al. 2019), and downstream helpers for primer extension (Prywes et al. 2016).

Finally, the spontaneous cleavage of longer RNA strands into shorter oligonucleotides would also interrupt oligonucleotide growth, but in a way that could still contribute to overall replication. Such strand cleavage events are most likely to occur during periods of elevated temperature when most or all of the RNAs are unpaired single strands, and would be catalyzed by Mg^2+^ and other divalent ions (Soukup and Breaker 1999). The consequences of strand cleavage events are somewhat complex, as the different cleavage products will have different fates and roles. When an oligonucleotide is cleaved, an upstream or 5′ product and a downstream or 3′ product are formed. The downstream product, with a 5′-hydroxyl and a 2′,3′ cis-diol, will simply reenter the pool of genomic oligonucleotides, and would participate in chain growth processes normally (except that, lacking a 5′-phosphate, it could not ligate to an upstream oligonucleotide). In contrast, the upstream fragment will initially terminate in a 2′–3′ cyclic phosphate, which cannot be extended by either primer extension or ligation reactions. However such a fragment could act as a template if long enough, or as an invader strand in RNA catalyzed strand displacement synthesis (Zhou et al. 2019). Subsequent hydrolysis of the cyclic phosphate will generate either a 2′ or a 3′-phosphate. Strands terminating in a 2′-phosphate remain inactive for primer extension (and probably for ligation), but can still act as templates or invaders. However, strands terminating in a 3′-phosphate can grow by primer extension or ligation, but in the process will generate a pyrophosphate linkage. Continued extension generates longer oligos with an internal 3′–5′-pyrophosphate linkage, which is extremely labile in the single stranded state. Pyrophosphate cleavage occurs by the intramolecular attack of the adjacent 2′-hydroxyl. This in turn generates a downstream fragment that becomes a normal genomic oligonucleotide, while the upstream fragment once again ends in a 2′,3′-cyclic phosphate, which could then reenter the processes just described (Wright et al. 2019).

## IMPLICATIONS OF THE CONCENTRATION VERSUS LENGTH PROFILE FOR GENOME REPLICATION

If replication of the virtual circular genome is fed largely by an input of monomers, possibly supplemented by smaller amounts of di- and trinucleotides, and if oligonucleotide elongation is inefficient, then short oligonucleotide fragments should be more abundant than longer oligonucleotides. Making the simplifying assumption that the concentration ratio for oligos of length n and n + 1 is independent of length defines a length versus concentration gradient of oligonucleotides within a protocell. This length distribution has an important implication for the replication of the genome as a whole, in that the steeper the gradient, the less oligonucleotide growth (by primer extension or ligation) is required to replicate the genome. For example, with an n/(n + 1) length versus abundance ratio of 2, the elongation of every oligonucleotide by only one nucleotide on average is sufficient to replicate the entire genome, as illustrated in Table 1. A shallower abundance ratio, for example an n/(n + 1) abundance ratio of $\sqrt{2}$ would require (or would result from) an average growth of each oligo by two nucleotides per replication cycle.

The possibility of achieving genome replication by extending oligonucleotides by only one nucleotide on average per cell cycle is a remarkable and unexpected consequence of the virtual circular genome model. Since highly efficient template-directed primer extension by up to seven nucleotides has been experimentally demonstrated using 2AI activated ribonucleotides as substrates (Li et al. 2017), it may well be possible to experimentally demonstrate this mode of nonenzymatic genome replication. The main limiting factors in achieving an oligonucleotide extension by one nucleotide on average are likely to be the probability that a given oligonucleotide will pair with others in a productive manner, and the probability that such a productive pairing will last long enough to allow primer extension or ligation to occur. In addition, both the input monomers and the genomic fragments would have to be maintained in an activated state (e.g., as a 5′-imidazolide) so that primer extension and ligation could continue to occur in the face of ongoing hydrolytic reactions. The recently described Sutherland pathway for activation through isocyanide chemistry provides one possible route to the continued maintenance of the activated state (Mariani et al. 2018b), although other simpler approaches may yet be uncovered.

A number of factors may influence the length versus concentration gradient during continued replication. For example, short oligonucleotides bind more weakly to templates than longer oligonucleotides, and as a result may be less likely to become extended if their productive pairing configurations are shorter lived. In addition, shorter oligonucleotides will cyclize more rapidly than longer oligonucleotides, and may thus be removed from the pool of replicating oligonucleotides more often. On the other hand, longer oligonucleotides will be subject to faster removal from the pool of replicating oligonucleotides due to degradation or the formation of thermally stable duplexes as discussed above. The net effect of these and possibly other factors on the concentration versus length gradient is unclear.

Because the melting temperature of an oligonucleotide duplex is a function of both concentration and length, a steep concentration versus length gradient would slightly flatten the slope of the *T*_m_ versus length gradient. For modern RNA, where each additional base-pair contributes ∼2–3 kcal/mol to duplex stability, the 0.3 kcal/mol effect of a twofold increase in concentration per additional nucleotide in length would have a relatively small effect. However, it is worth noting that some proposed prebiotic nucleic acids are expected to have much weaker base-pairing. For example, replacing G with I would weaken base-pairing (Wright et al. 2018), and the random mixing of deoxynucleotides with ribonucleotides in a chimeric polymer would weaken duplex stability even more (Bhowmik and Krishnamurthy 2019). In the extreme, a constant duplex *T*_m_, independent of length, might result. In this case at or near the *T*_m_, continuous reshuffling of paired configurations might occur, so that primer extension and ligation events could continue indefinitely without thermal fluctuations.

## EMERGENCE AND STABILITY OF VIRTUAL CIRCULAR GENOMES

How could protocells containing replicating virtual circular genomes arise? Here we discuss two possible scenarios. In the first, which we consider more likely, protocells would initially assemble in an environment with a high concentration of random sequence oligonucleotides. For example, in a shallow pool subject to wet-dry cycles, membranes and oligonucleotides could dry down together, after which wetting would result in the formation of vesicles containing a high concentration of RNA (Ross and Deamer 2016). Subsequent cycles of denaturation and reannealing would then enable oligonucleotide elongation as discussed above. We suggest that virtual circular genomes might emerge spontaneously in such a situation, because of the self-reinforcing autocatalytic nature of exponential genomic replication. If true, this might be an example of the long sought spontaneous emergence of self-organizing autocatalytic sets (Dyson 1982; Farmer et al. 1986; Kauffman 1986). Alternatively, inefficient and stochastic oligonucleotide elongation events might continue indefinitely without convergence. Only experimental tests can address this question.

There is a second way in which protocells might emerge with encapsulated virtual circular genomes. In this scenario, unencapsulated oligonucleotides could be generated either on mineral surfaces or under eutectic concentration conditions. Following 5′-phosphate activation, circularization could occur, albeit inefficiently for longer oligonucleotides. The encapsulation of such physically circular oligonucleotides within vesicles fed with activated monomers could lead over time to the synthesis of overlapping sets of linear oligonucleotides representing the complementary strand. These oligonucleotides could then act as templates to initiate the synthesis of the full set of oligonucleotides representing a virtual circular genome.

Could virtual circular genomes be stably inherited over many generations of protocell growth and division? This might seem to be a serious problem, since protocells by definition lack any evolved machinery to control the distribution of genetic material to daughter cells during division. However, if the genomic ensemble of oligonucleotides is present at a high average copy number, the purely statistical segregation of genomic fragments into daughter protocells could confer high stability. Depending on the copy number, the simultaneous loss of all oligonucleotides covering a region of the genome could be quite rare, except during the earliest stages of the emergence of a virtual circular genome when average copy number was low. If genome duplication during those early stages occurred more frequently than protocell division, high copy number would be rapidly attained, and the genome would be reasonably stable thereafter. Although the steady state genomic copy number would depend on many factors, a high copy number does not seem unreasonable, and we can make a very rough estimate of copy number as follows. First, assume that activated monomers and imidazolium bridged dinucleotides are present at the lower range of concentrations required for efficient primer extension (5 mM and 1 mM, respectively), that phosphodiester bonded di- and trinucleotides are present at similar concentrations (1 mM and 0. 5 mM). If we then assume an n/(n + 1) oligonucleotide concentration ratio of 2, as discussed above, it is then trivial to calculate the concentrations of all longer oligonucleotides (Table 2). Under these assumptions, 12-mer oligonucleotides would be present at a concentration of 1 µM, corresponding to a copy number of ∼10^6^ in a 10 µm diameter protocell, or 10^3^ in a 1 µm diameter protocell. Such copy numbers would be more than sufficient to confer stable genome inheritance given random segregation into daughter protocells.

**TABLE 2.**
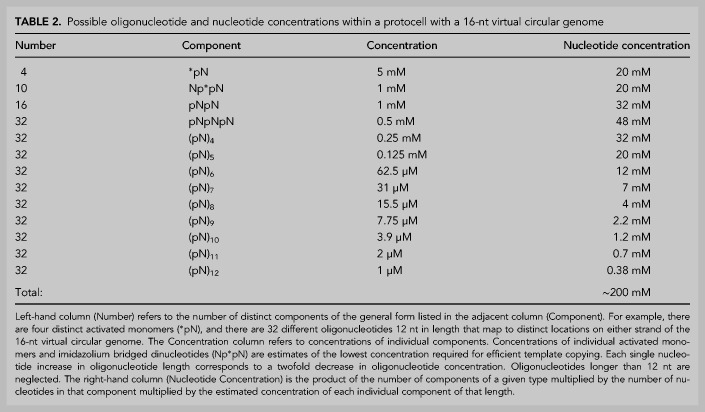
Possible oligonucleotide and nucleotide concentrations within a protocell with a 16-nt virtual circular genome

Some hard to replicate sequences might in effect break the circle as a result of a decreased copy number of oligonucleotides representing that region. However, mutations that render all regions of the circle similarly replicable would be selected for as a result of increased replication efficiency. A more serious problem would arise if the error frequency of the template-directed oligonucleotide elongation reactions was too high. In addition, mispairings as a result of chance complementarity between oligonucleotides from different parts of the circular genome could result in incorrect primer extension events. If genomic integrity could not be maintained, the oligonucleotide ensemble could diverge to a set of random sequence fragments. Since chance mispairings would become more common in larger virtual circular genomes, such events might impose an upper limit on the size of virtual circular genomes.

What prebiotic geochemical environments might support RNA replication via the virtual circular genome model? The model suggests two likely constraints. First, multiple thermal or other fluctuations that would allow the shuffling of base-paired configurations would probably be necessary for each duplication of the RNA genome of a protocell. Second, thermal fluctuations in particular must be brief, so that single-stranded RNA oligonucleotides are not exposed to high temperatures for extended times, which would lead to excessive degradation. Experimental models of such fluctuating environments have been studied extensively by Braun et al. (Ianeselli et al. 2019; Morasch et al. 2019; Salditt et al. 2020). Volcanic or impact related hydrothermally active settings have the potential to provide such rapidly fluctuating environments in nature. Hot springs, mud pots, geysers and fresh water hydrothermal vents all provide steep and variable temperature gradients in local settings, on time scales much faster than day-night cycles. Such environments are frequently invoked as possible sites of wet-dry cycles (Ross and Deamer 2016; Damer and Deamer 2020), and we suggest that localized regions might also provide the rapidly fluctuating environmental parameters required to support RNA replication by the virtual circular genome model.

## ASSEMBLY OF RIBOZYMES FROM THE VIRTUAL CIRCULAR GENOME

The largest oligonucleotides in a virtual circular genome are likely to be on the order of 10 to 12 nucleotides in length, because longer oligonucleotides would likely form overly stable duplexes. Such oligonucleotides are probably too short to act as efficient ribozymes in isolation. Whether such oligonucleotides could noncovalently assemble into active ribozymes is unclear. On the one hand, such relatively short oligonucleotides could not form stable base-paired stems flanking single-stranded regions, limiting the complexity of noncovalent assemblies. On the other hand, it is possible that very simple assemblies might exhibit selectively advantageous functionality.

How might longer oligonucleotides, more suitable for assembly into complex and highly active ribozymes, arise? A likely possibility is that multiple RNA oligonucleotides could be covalently joined by repeated ligation steps. For example, we have recently shown that the splinted ligation of five 10–12 nt long oligonucleotides can efficiently generate an active ribozyme ligase that is 52 nt in length (Zhou et al. 2020b). However, the splint oligonucleotides used to template the ligation were strong inhibitors of the ribozyme, unless their affinity for the ribozyme sequence was reduced. One of the simplest and most prebiotically plausible means of achieving the necessary combination of high specificity and low affinity was by replacing guanosine with inosine. We have previously suggested that inosine is at least as good and possibly superior to guanosine in terms of template copying efficiency and fidelity (Kim et al. 2018). Thus, a primordial genome containing inosine in place of guanosine would seem to have multiple advantages, including more efficient replication and the ability to generate long oligonucleotides with ribozyme activity.

It has long been known that pyrimidine rich oligonucleotides are superior to purine rich strands as templates for chemical copying by activated nucleotides (Joyce and Orgel 1986; Joyce et al. 1984), presumably because of the greater stacking energy available to stabilize the binding of activated purine ribonucleotides to a template strand, in a position adjacent to a primer. This bias in copying efficiency could therefore lead to a bias in the abundance of pyrimidine and purine rich oligonucleotides in the virtual circular genome model. In other words, if one strand or region of the circular genome is pyrimidine rich and the other is purine rich, one would expect the fragments of the purine rich strand to be overrepresented in the overall collection of oligonucleotides. Interestingly, the unpaired regions of aptamers and ribozymes also tend to be enriched in purines (Patel et al. 1997; Kennedy et al. 2010). Thus, an excess of purine rich oligonucleotides could favor the assembly of ribozyme sequences, while the limited abundance of complementary pyrimidine rich oligos would decrease the problem of ribozyme inhibition by complementary splints.

## EVOLUTION OF VIRTUAL CIRCULAR GENOMES

Primitive protocells would be essentially at the mercy of their environment for the specialized sources of energy, molecular building blocks, and highly specific conditions required for growth, division and replication. As a result they would be subject to extremely strong selective pressures favoring the evolution of catalysts, structures and regulatory mechanisms that would provide enhanced survivability and reproductive success as well as the ability to adapt to different or changing environments and thus to colonize new ecological niches. The nature of the first ribozymes to evolve is a highly speculative topic. Indeed, the possibility of watching the evolution of new ribozymes in real time is one of the primary motivations for devising laboratory populations of replicating model protocells. Although much emphasis has been placed on the evolution of RNA replicase ribozymes, it is not clear that RNA replication per se would be selected for in a population of protocells (Szostak et al. 2001; Szostak 2017). In contrast, it has been experimentally demonstrated that changes in membrane composition can have strong effects on vesicle growth. In particular, a ribozyme that catalyzed the synthesis of either two-chain phospholipids or hydrophobic peptides could confer a strong growth advantage to protocells with fatty acid based membranes (Budin and Szostak 2011; Adamala and Szostak 2013). Once such a ribozyme had evolved, there would then be a strong selective pressure favoring the evolution of new catalytic activities that would result in more efficient and accurate replication of the sequences encoding the first ribozyme. Thus, in short order, the virtual circular genome(s) of the first protocells might be replaced by more sophisticated RNA genomes replicated by RNA polymerase ribozymes.

A potential problem with the emergence of the first ribozymes is that ribozymes tend to be much less active than modern highly evolved protein enzymes. Having a selectively advantageous effect on a protocell may therefore require a significant concentration of low-activity ribozyme molecules. For example, a ribozyme concentration of ∼0.1 µM in a 10 µm diameter protocell might be necessary to catalyze a reaction at a rate that is useful to the protocell. A protocell of that size would contain ∼2 × 10^9^ lipid molecules in its membrane; for a ribozyme to modify 5% of these molecules, at a turnover rate of 1 min^−1^, and a one day (10^3^ min) generation time would require ∼10^5^ ribozyme molecules. Whether so many ribozyme molecules could be assembled in each generation is unclear.

Multiple factors might affect the evolvability of protocells with virtual circular genomes. A high copy number of the component oligonucleotides of a virtual circular genome implies that randomly arising mutations would initially be at such a low concentration that their potentially positive or negative effects would not be manifest. However, random segregation of wild type and mutant sequences would rapidly lead to protocells in which mutant sequences had either been eliminated or fixed (Szostak et al. 2001), as seen in modern cells bearing high copy number plasmids or arrays of ribosomal RNA genes (Ganley and Kobayashi 2011). A more important parameter is likely to be the fidelity of nonenzymatic replication. In order to avoid an Eigen error catastrophe, replication must proceed with an error rate that is roughly the reciprocal of the genome length (Eigen and Schuster 1977). Whether chemical replication can attain sufficient accuracy remains to be determined.

## EXPERIMENTAL TESTS OF THE VIRTUAL CIRCULAR GENOME MODEL

Even small virtual circular genomes are represented by collections of hundreds of oligonucleotides. This complexity poses significant experimental challenges, from both a synthetic and an analytical point of view. The simplest, but most tedious and expensive way to synthesize examples of virtual circular genomes would be to prepare each oligonucleotide separately, and then mix the oligonucleotides in the proportions corresponding to the desired concentration versus length gradient. Such mixtures could then be encapsulated in lipid vesicles for protocell replication studies. Alternatively, subsets of oligonucleotides could be prepared and then mixed; this approach would be less expensive but would come at the cost of some analytical uncertainty about the composition of the final pool of oligonucleotides.

Once appropriate mixtures of oligonucleotides have been prepared, replication chemistry must be studied under a range of environmental conditions, while feeding with different sets of activated monomers and oligonucleotides in the presence of different forms of in situ activation chemistry. Unfortunately, no current technology allows for the simultaneous evaluation of the fates of all of the diverse oligonucleotides present in each starting pool. Polyacrylamide gel electrophoresis and even the most advanced mass spectrometry methods lack the resolving power to follow all of the oligonucleotides in parallel. This problem is exacerbated by the large concentration of differences between the shortest and longest oligonucleotides in the pool. However, the fate of individual oligonucleotides could easily be followed by the synthesis of separate radio-labeled oligonucleotides which could be added to the total ensemble as tracers. Such experiments could determine the effective rates of primer extension, ligation and fragmentation for individual oligonucleotides, and could allow the development of a global picture of replication over multiple experiments. An alternative approach that would provide complementary information would be to modify current methods for deep sequencing of RNAs, so that the relatively short oligonucleotides of virtual circular genomes could be directly sequenced. This approach could provide both quantitative information on changing oligonucleotide frequencies, and, more importantly, information about the fidelity of the replication process and even the evolution over time of the oligonucleotide ensemble. Ultimately, a combination of measurements of the concentrations of oligonucleotides of different lengths, together with sequence information, should allow for evaluation of the laboratory replication of RNAs through nonenzymatic chemical processes.

## CONCLUSIONS

The virtual circular genome model differs from previous models in that it does not involve the synthesis of a complete copy of one genomic template molecule; instead, genome replication is a distributed process in which an ensemble of genomic fragments grow by as little as one nucleotide per genome doubling through template-directed reactions, but with each fragment elongating on a different template molecule. Over multiple generations, this mode of genomic replication can be viewed as a flow of material from short initiating oligonucleotides through progressively longer oligonucleotides all of which map onto the sequence of the virtual circular genome. This model resolves many of the problems that have until now plagued efforts to formulate viable models for the nonenzymatic replication of primordial RNA genomes. The model obviates the need for specific primers, renders the “last base addition problem” irrelevant, and avoids the need to copy long RNA templates within a single generation. Importantly, this mode of replication is testable via straightforward laboratory experiments. If successful, such experiments could lead to the facile construction of replicating, evolving model protocells, which could in turn provide further insight into key steps in the origin of life.
